# Patient and public involvement to build trust in artificial intelligence: A framework, tools, and case studies

**DOI:** 10.1016/j.patter.2022.100506

**Published:** 2022-06-10

**Authors:** Soumya Banerjee, Phil Alsop, Linda Jones, Rudolf N. Cardinal

**Affiliations:** 1 University of Cambridge, Cambridge, UK; 2 Cambridge, Cambridgeshire, UK

## Abstract

Artificial intelligence (AI) is increasingly taking on a greater role in healthcare. However, hype and negative news reports about AI abound. Integrating patient and public involvement (PPI) in healthcare AI projects may help in adoption and acceptance of these technologies.

We argue that AI algorithms should also be co-designed with patients and healthcare workers.

We specifically suggest (1) including patients with lived experience of the disease, and (2) creating a research advisory group (RAG) and using these group meetings to walk patients through the process of AI model building, starting with simple (e.g., linear) models.

We present a framework, case studies, best practices, and tools for applying participative data science to healthcare, enabling data scientists, clinicians, and patients to work together. The strategy of co-designing with patients can help set more realistic expectations for all stakeholders, since conventional narratives of AI revolve around dystopia or limitless optimism.

## The bigger picture

Hype and negative news reports about artificial intelligence (AI) abound. Involving patients in healthcare AI projects may help in adoption and acceptance of these technologies. We argue that AI algorithms should be co-designed with patients and healthcare workers.

We show examples of how to involve patients in AI research and how patients can build trust in algorithms. We share some best practices, case studies, a framework, and computational tools.

Avenues for future work include guidelines for patient and public involvement in AI healthcare research for funding bodies and regulatory agencies.

An understanding of what AI can and cannot do, and a realistic appraisal of risks and benefits, may help in adoption and democratize access to AI for healthcare.

## Teaser

We argue that AI algorithms should be co-designed with patients and healthcare workers.We suggest including patients with lived experience of the disease, and creating a research advisory group (RAG) and using these group meetings to walk patients through the process of AI model building, starting with simple (e.g., linear) models.We present case studies, best practices, tools, and a methodology of how collaborative data science can be applied to healthcare, where data scientists, clinicians, and patients work together.

## Introduction

Machine learning is increasingly becoming pervasive in healthcare. Artificial intelligence (AI) is increasingly taking on a greater role in healthcare, especially during the current coronavirus disease 2019 (COVID-19) pandemic.^1^ However, hype and negative news reports about AI abound.

People do not always understand or trust AI. This overlaps with other concerns people have, such as the security of their data. People are not always consulted about AI that might affect them. Part of the solution is patient and public involvement (PPI). In PPI, the general public and patients are involved in research. The level of involvement varies from project to project. Being involved in a project helps build trust. There is a rich history of PPI in healthcare. However, it has not been done very much in the context of modern AI.

As misinformation spreads around AI, integrating patient and public involvement in healthcare AI projects and clinical trials may help in adoption and acceptance of these technologies. We argue that AI software should also be co-designed with patients and that patients should be involved in discussions around AI research applied to healthcare.

We advocate a collaborative approach where patients, carers, clinicians, and data scientists work together to decide what data will be used as inputs to computer programs and understand why these algorithms made a particular prediction. Recent studies have raised awareness about designing AI algorithms in close collaboration with healthcare workers.^1^ Machine learning researchers alone may not be able to appreciate the broader impact of their work and there is a need to involve other stakeholders.^2^

We give examples of work we have done in this area as case studies (in the section “case studies: examples of data-focused research via a research advisory group”), and make some general recommendations (in the sections “framework for building trust and typical patient concerns” and “recommendations”). We suggest a framework of how patients can build trust in AI and we share tools and resources that can be used to explain the basics of AI to patients. We developed tools to demonstrate key concepts to the public (section “tools for outreach and involvement”). We also review the current literature on trust in AI (section subsection “trust in AI and the role of PPI”). We hope that the approach of involving patients, clinicians, and data scientists in a virtuous cycle of co-design will be used in future AI projects in healthcare.

## Case studies: Examples of data-focused research via a research advisory group

In this section, we describe two projects as case studies. In later sections, we reflect on these projects and present our general recommendations.

We were conducting research in this area, so we recruited patients and formed a research advisory group (RAG). The RAG met regularly and discussed data-focused research projects related to severe mental illness. Additional details on the RAG are available in the section “framework for building trust and typical patient concerns.”

### Analysis of the effect of lithium medication on kidney function

In this section we describe a patient-led project. Patients with bipolar disorder are sometimes prescribed lithium. Lithium is an effective medication, but long-term use may lead to kidney damage. A patient in the RAG had suggested looking at hospital data to investigate if discontinuing lithium can help recover kidney function in patients with bipolar disorder taking lithium.

The patient was involved in all stages of a research project. Our aim was to predict whether stopping lithium intake, in patients with bipolar disorder (who have been on the medication for a long time), is associated with reversal of drug-induced renal damage.

We used observational from hospital electronic healthcare records systems to answer these questions. We outline the various datasets that were used in this work:

(1) EPIC prescription data. This is an electronic patient record system operational in Cambridge University Hospitals (CUH) from October 2014 until present. This system captures all CUH activity during its period of operation. This includes laboratory tests and prescriptions (these are recorded typically for inpatients only) and structured diagnostic codes (for a subset of patients and a subset of diagnoses). This has features like age, gender, and ethnicity.

(2) Meditech data. This is a laboratory system operational in CUH from 1995 until present. This system captures all laboratory investigations data from CUH during its period of operation. This has laboratory results like creatinine.

Patients with records in both EPIC and Meditech had their records cross-matched before anonymization.

We used the following linear mixed effects model (in the R programming language notation):

(Equation 1) $$e_{GFR}=e_{0}+b_{off}t_{off}+b_{on}t_{on}+\left(1|pid+t_{off}+t_{on}\right)$$

where $e_{GFR}$ is the estimated glomerular filtration rate (eGFR) and is calculated from creatinine, age, gender, and ethnicity (data available from hospital electronic healthcare records system) using the CKD-EPI formula.^3^ pid is the unique patient identification number in the electronic healthcare record system. $b_{on}$ is the rate at which eGFR declines when a patient is on lithium. $t_{off}$ is the cumulative time spent off lithium, and $t_{on}$ is the cumulative time spent on lithium. $b_{off}$ is the rate at which eGFR is declining for patients off lithium, and $b_{on}$ is the rate at which eGFR is declining for patients on lithium. $e_{0}$, $b_{on}$, $b_{off}$, $t_{on}$, and $t_{off}$ are parameters that are estimated from the data.

However, using these data on a few thousand patients, the results were inconclusive. This motivated the need to go back to the RAG and explain the need for more data. We took feedback from patients as to whether we should apply for access to more data. We also built a tool that explains how, in some cases, having more data can help in estimating parameters of statistical models (see sections “tools for outreach and involvement” and “framework for building trust and typical patient concerns”). This process of performing research and getting inconclusive results also showed patients how research always takes time and can lead to unexpected roadblocks.

We also took the time to explain how to build statistical models. For example, we tried other, simpler formulations before we arrived at the final model (see Equation 2). This showed patients how researchers always incrementally build more complex models.

(Equation 2) $$e_{GFR}=e_{0}+b_{off}t_{off}+b_{on}t_{on}+\left(1|pid\right)$$

We explained these models using an example of a simpler linear model:

(Equation 3) y=a⋅x+b

This is a linear model where the value of *x* is used to predict *y* (say eGFR). *a* and *b* are the parameters of the model, and these can be estimated. We explained that estimating means determining the values of *a* and *b* from data. Once we explained the concepts of a linear model, we progressed to more advanced concepts like confidence intervals.

We are currently validating our results in an additional independent cohort of patients (the Clinical Practice Research Datalink [CPRD] research database, which has general practice records from the United Kingdom^4^).

At this stage, we also communicated to the patients a number of caveats. Lithium is an effective medication for managing bipolar disorder,^5^ and the chances of patients developing renal complications is quite small.^6^ The benefit of discontinuing lithium should be carefully weighed against the risk of relapse of the psychiatric disorder, as has been documented in case studies^7^ and suggested in meta-analysis studies.^6^

Our work may lead to randomized controlled trials to test the hypothesis that discontinuing lithium may help recover kidney function in patients with bipolar disorder.

## Predicting mortality in patients with severe mental illness

Premature mortality in patients with severe mental illness (like schizophrenia) is a public health concern. Here we outline another project where we used observational data from electronic healthcare records to make predictions of mortality in patients with schizophrenia.

We developed machine learning models of mortality in schizophrenia and applied the technique of class-contrastive reasoning to improve their explicability. Class-contrastive reasoning is a technique from the social sciences^8^^,^^9^: the contrast is to an alternative class of exemplars. An example of a class-contrastive explanation is a selected patient who is at high risk of mortality because the patient has dementia in Alzheimer’s disease and has cardiovascular disease. If the patient did not have both of these characteristics, the predicted risk would be much lower.

Briefly, the approach modifies the features until the machine learning model produces a different prediction. The effect of changing features on the model output is explained visually using a heatmap.^10^

The machine learning model was trained on the training data. We then changed one feature at a time on the test data and recorded the change in the model prediction. In this scenario, the model is not retrained. The change in model predictions was visualized as a class-contrastive heatmap.

For this project, the researchers defined the hypothesis. Discussions with patients then motivated the need to develop an explainable AI algorithm.^10^

Our machine learning approach^10^ is summarized here:

1 We used de-identified data from an electronic patient record system for mental health.
2 We defined a set of high-level features These included age, psychiatric diagnostic categories (time-independent coded diagnosis at any point during the study period), and medication categories (time-independent prescription of or use of medications; for example, anti-depressants). We also included bio-social factors that are important in severe mental illness, like information on mental health diagnosis, relevant risk history such as a prior suicide attempt, substance abuse, and social factors such as lack of family support.
3 We used these features to predict death during the time of observation.
4 We then fitted a machine learning model. Our machine learning algorithm was based on a type of artificial neural network called an autoencoder.^10^ Class-contrastive heatmaps were used to visualize the explanations of the statistical models and machine learning predictions. The corresponding class-contrastive statements and heatmaps also aid human interpretation.

We used data from the Cambridgeshire and Peterborough National Health Service (NHS) Foundation Trust (CPFT) Research Database. This comprises electronic healthcare records from CPFT, the single provider of secondary care mental health services for Cambridgeshire and Peterborough, UK, an area in which approximately 856,000 people reside. The records were de-identified using the CRATE software^11^ under NHS Research Ethics approval (12/EE/0407, 17/EE/0442).

Data included patient demographics, mental health, and physical co-morbidity diagnoses: these were derived from coded International Classification of Diseases, tenth Revision (ICD-10) diagnoses and analysis of free text through natural language processing (NLP) tools.^12^^,^^13^

Dates of death were derived from the NHS Spine. We considered all patients with coded diagnoses of schizophrenia who had records in the electronic healthcare system from 2013 onwards. There were a total of 1,706 patients diagnosed with schizophrenia defined by coded ICD-10 diagnosis (diagnosis code F20).

We note that our machine learning and statistical models are not meant to aid decision making in their present form. Additional validation studies in other cohorts and evaluations will be required to determine if these models can be used in clinical decision making.

## Framework for building trust and typical patient concerns

In this section, we outline a framework for building trust in AI. We share some of the typical concerns that patients have about AI and how to address them. The nature of explaining and understanding a complex model (like an AI model) requires humans to build simplified mental representations.^14^ Trust in an AI system can be built up slowly after understanding it at multiple levels, ranging from personal to institutional and technological.^15^

In order to build trust in AI algorithms, one needs to consider the complex socio-technological milieu in which technological solutions reside. Trust needs to be built not only in AI algorithms, but the training data, software, and complex environment in which humans are situated. These include institutions and people.

The doctor-patient relationship is an important aspect of trust.^16^ Trust in institutions and people is intimately linked to trust in health technologies.^15^

Our patients may have had implicit faith in the institutions where this research was conducted (Cambridge University Hospitals and University of Cambridge) and the carers involved (L.J., R.N.C.)). We note that this can be very difficult to achieve in places where there is an existing trust deficit in doctors and hospitals, especially in low- and medium-income countries.^17^

This step is probably the most difficult to implement and the least actionable from the viewpoint of an individual researcher who is embedded in a large organization. Nevertheless, we wish to point out that trust in algorithms cannot be completely decoupled from trust in institutions and people involved in the research.

For existing organizations that have built trust and reputation over decades, this is quite simple. For emerging institutions, especially in developing nations, this is a very big challenge. Building trust and reputation takes decades of work. This is not to under-emphasize the importance of algorithms but to merely point out the complex socio-technological milieu in which all technological solutions reside.

We outline a framework below for helping patients build trust in AI. We note that there is no particular order to this framework.

1 Recruiting patients and forming an RAG.

We recruited patients and formed an RAG. We designed an advertisement explaining the project and a person role profile, which was sent out through our local long-standing PPI groups across the region. The requirements were to have a lived experience of mental health illness or care for someone who does. Once the applications came in, L.J. went out and met everyone to explain further about the group and to answer any questions people may have had. Out of the nine original applicants, five became fully involved as part of an RAG and one was involved from a distance (i.e., did not meet up with the group but was happy to give advice from a distance).

The RAG were then invited to a meeting to tell them about the team projects. We then kept in touch through further emails, calls, and occasional meetings (although the in-person meetings were curtailed with the onset of the COVID-19 pandemic).

The RAG also co-produced questions in surveys that were sent out to patients. We helped them understand what we hoped to get out of the project and they then helped formulate the questions needed to get the answers and helped design surveys.

For recruitment to the survey study, we advertised in many ways through written posters and social media as this was a survey for both patients and public. We also recruited through over 100 physical and mental health trusts through the UK National Institute for Health Research (NIHR) Clinical Research Network (CRN). Sites were not specifically selected: through the CRN, we took on board any site who wanted to recruit for us. They approached their patients in person, staff, newsletters, social media, television screens in clinics, posters, PPI groups, users networks, and so forth. We also approached over 200 general practice surgeries across the country, who contacted patients by text, newsletters, social media, posters, and television screens.

A kick-off meeting was organized when the RAG was constituted. RAG meetings with all team members were then held once every 6 months over a 2-year period. More focused meetings on projects (see section “case studies: examples of data-focused research via a research advisory group”) were held approximately once in 2 months.

2 Problem formulation and hypothesis generation.

Scoping and framing the problem is very important.^18^ Some problems are relevant but cannot be answered with the data we have. It is important to determine the intersection of a relevant problem, and having the data and expertise to solve it. In the initial discussions with the RAG, we determined and scoped two research questions, which can be addressed using data we had access to. These are detailed in the section “case studies: examples of data-focused research via a research advisory group.”

One project focused on the effect of a medication (lithium) on kidney function (section “case studies: examples of data-focused research via a research advisory group”). The patient formulated the problem and initial hypothesis: stopping lithium intake (a drug prescribed for bipolar disorder that can in some cases cause renal toxicity) may reverse renal damage.

The patient also suggested potential roadblocks like non-adherence of lithium medication. For example, some patients do not take lithium consistently. Instead, a few days before a lithium blood test, they take the medication (so that lithium is detected in a blood test). It is something we had not appreciated before. The patient suggested this and we took steps to computationally account for this non-adherence.

Another project focused on data available from hospital electronic healthcare records (EHRs) and used it to predict mortality in patients with severe mental illness. Motivated by discussions with the RAG, we decided to build an interpretable machine learning model for this problem^10^ (see sections “case studies: examples of data-focused research via a research advisory group” and “predicting mortality in patients with severe mental illness”).

3 Building trust in the data storage infrastructure.

Concerns about data privacy and the fear of data exploitation are impediments to adoption of digital health technologies.^15^ We explained our data storage infrastructure to patients. We explained that all clinical data were stored on computers in a secure environment that was part of the NHS. Researchers had to apply for research passports to get access to the data, and all analysis had to be performed on those secure computers. We explained that obtaining a research passport involved detailed background checks and only eligible researchers could get access to the computational and data storage infrastructure.

All data were also pseudonymized, which made it extremely difficult to identify individual patients. We took care to explain that although the possibility of identification was minimal, no system was secure against a determined adversary. The data scientist also faced various roadblocks and administrative delays in data access, which only served to demonstrate the considerable difficulties that an adversary would have to overcome.

4 Addressing concerns about big data.

Patients may also have concerns about big data and their data being used. These concerns may relate to storing large amounts of data and whether it posed any risk for privacy.

In order to address this concern, we explained the steps we are taking to ensure patient privacy. For example, we explained how all data were stored securely on NHS computers, which only eligible researchers had access to. The process of getting access to the data included a lengthy research passport application (which also involved a background check).

We also elicited feedback from patients as to whether we should have access to additional data. For the project on lithium-induced renal toxicity (section “case studies: examples of data-focused research via a research advisory group,” subsection “analysis of the effect of lithium medication on kidney function”), we determined that we needed follow-up data for patients or access to a larger external cohort of patients in CPRD.^4^

Before requesting access to CPRD data (unlinked and anonymized),^4^ we asked for the opinion of patients and explained that having more data may lead to better performance in our models.

As a simple example, we also demonstrated a computational tool that showed the advantages of using big data in healthcare^19^ (see section “tools for outreach and involvement”). We also undertook surveys to understand more broadly the concerns that patients have about using big data in healthcare.

5 Discrimination and bias in AI.

Discrimination and bias is a valid concern for patients. We showed how this can happen in a simple situation of a facial recognition tool^20^ (see section “tools for outreach and involvement”). For example, if a training dataset has no data on faces of people from a certain ethnic background, then the machine learning algorithm implementation being trained to recognize faces will not have “seen” these data before.^21^ Hence when this machine learning algorithm implementations is used to make a prediction, it may be biased against these individuals.^22^

We showed how we are taking precautions against bias in algorithms and the data. We stressed the fact that the tools that we build will ultimately need to be validated in another setting with more patient numbers. We explained that sensitive data like date of birth, home addresses, and NHS numbers will not be stored. Sensitive attributes contribute toward perceptions of fairness in data.^23^

Data can mirror historical societal biases. A critical examination and discussion of bias in data, may allow us, including patients, to re-envision a future where AI is used for good.^22^

6 Debunking myths about AI from contemporary discussions in the media.

Patients may have misconceptions about AI from discussions in the media. An online resource^24^ (also see section “tools for outreach and involvement”) can be used to debunk common myths surrounding AI.

7 Understanding a simplified model.

We simplified the problem and built a simple linear model (the model is explained in detail in the section “case studies: examples of data-focused research via a research advisory group,” subsection “analysis of the effect of lithium medication on kidney function”). We explained the basics of fitting a linear model to data and then explained the predictions of this simple model. Following this, we explained the motivations for fitting more complex models. Understanding simple linear models also built the foundation for more complex models like deep learning. This also helped patients understand how models are always built iteratively, by progressively adding more complexity.

Furthermore, we used heatmaps to visualize data and the output of models. We explained how heatmaps can be used to explore complex AI models, by visualizing how the model output changes when the input is changed^10^ (see section “case studies: examples of data-focused research via a research advisory group,” subsection “predicting mortality in patients with severe mental illness”).

8 Understanding AI models more broadly.

We used tools to help patients understand the basics of AI and deep learning. The Teachable Machine^25^ is one of these tools that can help patients understand deep neural networks by training and visualizing AI models in a Web browser.

We also built a tool to demonstrate the benefits of using big data in healthcare.^19^ Patients can play around with these tools and build an understanding of these models (see section “tools for outreach and involvement” for more on these tools).

9 Designing computer interfaces with patients.

As an additional step, computer interfaces can also be co-designed with patients. An example of this is the development of a smartphone app that was co-designed with patients.^26^

We had a long and deep engagement with patients in all steps of AI research, from hypothesis generation to model building and understanding. In this way, patients felt they were involved in this project. This also gave a sense of agency and voice to patients (see section “patient perspective”).^27^

## Recommendations

We specifically suggest (1) including patients with lived experience of the disease and carers, (2) creating an RAG and using these group meetings to involve patients and carers in all stages of the scientific process (starting from hypothesis generation). We also recommend explaining the process of AI model building, starting with simple (e.g., linear) models. We suggest using freely available AI models that run in the browser (such as the Teachable Machine^25^) to explain the basics of AI to patients. These meetings should be repeated to elicit feedback from the stakeholders, explain model predictions, and get guidance on model modifications.

In RAG meetings, we built trust and solicited comments on how patient data could and should be analyzed. We showed patients how we took precautions to preserve privacy and allayed other concerns. We sought to reduce the hype around AI and explain these techniques using simple examples.

We explained how AI will be used on clinical data and how the expected outcomes might benefit patients. In turn, we learned from patients and carers about important features of the data, and about the concerns that must be addressed to implement AI models in practice, including the potential for inadvertent discrimination by AI.^28^, ^29^, ^30^

We suggest a general framework of how patients can build trust in AI (see section “framework for building trust and typical patient concerns”). This framework can be adapted based on the unique requirements and financial constraints of a project.

## Patient perspective

Patients and carers have important research ideas about how best to improve quality of life, manage symptoms, offer existing treatments, or develop new interventions. Often these ideas differ from those prioritized by academia or the pharmaceutical industry.

Here is why two members got involved in research in our group:

“I decided to join the group to help make a difference using my experience with a mental health condition, in my case being diagnosed with bipolar in 2003. To offer and share ideas and tips in what helps me and also share my experience to help research in the future.”

“I was excited when I saw the invitation to join this group. Using more extensive data can potentially answer many vital questions that an 8-week drug trial simply cannot. As a service user I was keen to see how we can be involved.”

## Tools for outreach and involvement

There is a need for tools that help the public gain an understanding of AI. We outline some resources to demonstrate the basics of AI to general audiences and patients. All of these are freely available resources that can be demonstrated on a modern computer with an internet connection.

1 AI models that can be trained, run, and visualized in the Web browser, like the Teachable Machine.^25^

An effective way to build trust and understand a model is to actively construct it.^31^^,^^32^ Tools like the Teachable Machine lower the barrier to entry by training and visualizing AI models in a Web browser.

2 An AI model that runs in the Web browser and uses a webcam to detect facial expressions.^20^

This tool can be used to highlight the importance of a diverse training set. For example, if the training set does not have data on people of different ethnicities, then there is a risk of discrimination, because the model has not seen this kind of data before.

3 A Web application that demonstrates the benefits of big data in healthcare.^19^

Some patients may have concerns about collecting and using large quantities of data. This tool can be used to demonstrate that, for certain diseases, we may need more data, and more data may lead to better model predictions.

4 A set of videos that demonstrate what AI can and cannot do.^33^^,^^34^ These resources can help set patient expectations about AI.
5 A resource of myths about AI.^24^^,^^30^

Myths about AI abound. These resources^24^^,^^30^ can be used to debunk some of the myths surrounding AI.

## Engagement, involvement, and participation

There are different ways in which patients and carers can be involved and engaged. In this section, we outline different forms of engagement, involvement, and participation. Projects can adapt some of these based on their budget and time constraints.

Patient and public engagement is where information and knowledge about research is provided and disseminated. Examples of engagement are science festivals open to the public and research open days where members of the public are invited to find out about research. An understanding of AI and how it affects our society can also be effected through media, such as television programs, newspapers, and social media. Substantial costs may be involved in this.

Some resources outlined in the section “tools for outreach and involvement” can be used to demonstrate the basics of AI to patients and the general public. These resources can be run on a laptop with an internet connection and are a low-cost solution to raising awareness.

Even though we have released computational tools and these can be used by researchers worldwide, these are only one component of an engagement and patient involvement strategy. We had to actively collaborate and engage with patients. We anticipate this will be especially challenging in low- and medium-income countries, where researchers have limited budgets.

Patient and public participation is where people take part in a research study. For example, people can be recruited to a clinical trial or other research study to take part in the research. Patients can also be asked to complete a questionnaire or participate in a focus group as part of a research study. Some costs will be involved in recruiting patients and organizing meetings.

PPI is where members of the public are actively involved in research projects and in research organizations. Patients can be involved in identifying research priorities and formulating hypotheses. They can be joint grant holders or co-applicants on a research project. Patients and carers can also develop patient information leaflets and other research materials.

This is more complex and cost-intensive, and may require a project manager to organize groups, arrange meetings, and liaise with patients.

We suggest a framework (see the section “framework for building trust and typical patient concerns”) that shows how to engage with patients and how to adapt some of these techniques based on the unique budgetary constraints of a project.

## Discussion

Actively engaging patients in managing the illnesses that affect them may lead to more sustainable and patient-centered healthcare. This can be achieved by healthcare professionals working in co-production with patients and carers.

Lived experience of a disease is important in healthcare research.^35^ If properly designed, PPI can lead to better outcomes in health research.^36^ Considering the viewpoints of patients can also allow us to design future ethnographic studies and structured interviews for designing better AI solutions.^37^ Examining the unique relationships between patients and clinical staff in a healthcare organization may also help us design better electronic health record systems.^38^

### Caveats and limitations

This approach is not without limitations. Setting up and running PPI groups and meetings is a major undertaking. We had a dedicated project manager for PPI who is also qualified as a nurse (L.J.). Some projects may not have the resources for a project manager dedicated to PPI. It may be helpful to have PPI managers who lend their expertise to multiple research projects, thereby spreading costs across teams.^39^

Patient expectations also need to be managed by researchers.^36^ Patients may also not have the right quantitative skills, in which case it may be necessary to give them training or utilize their diverse complementary skills in other ways (see section “engagement, involvement, and participation”).

The patients who are recruited or volunteer for PPI group meetings may also not be representative of all patients. Crucially, the perspective of marginalized patient communities may be missed.

We believe the recommendations are supported by the extant literature (reviewed later in subsection “trust in AI and the role of PPI”). We note that a more thorough analysis of precisely which recommendations are most effective would require a human factors study. This may take the form of a randomized controlled trial or a formal change management study,^40^^,^^41^ in order to understand which steps in a roadmap lead to the most effective adoption of AI or trust in AI. This would require a formal quantitative study of which factors/steps work best. In this work, we rely on the extant literature and qualitative studies.^15^ We hope our work will inspire more quantitative recommendations on how to engage with patients.

We anticipate several hurdles for generalizing this work. We had a PPI lead (L.J.). L.J. is qualified as a research nurse and has spent many years caring for patients. R.N.C. is a clinician and also has experience applying statistical learning techniques to mental health. It is helpful if carers are also cross-trained in medicine and data science. Having researchers who are cross-trained in medicine and the quantitative sciences and/or have an appreciation of what data science can do in healthcare may be helpful in enabling successful PPI projects in AI.

We needed long-standing collaborations and research networks to recruit patients. We also had to actively collaborate and engage with patients. We anticipate this will be especially challenging in low- and medium-income countries, where researchers have limited budgets. Even though we have released computational tools and these can be used by researchers worldwide, these are only one component of an engagement and patient involvement strategy.

Engaging with stakeholders to define a problem sometimes leads to shallow definitions. It may also be difficult to find patients who are really interested in engaging with researchers. Patients may also lack quantitative skills or experience in research and may need training. Finally, we note that researchers are not incentivized to engage with patients.

In our experience, this framework is likely to succeed when patients really want to be involved and are curious about the research process. Research is also not a linear process, and communicating this to patients was a challenge. The framework is also not linear and may need to be adapted based on the idiosyncrasies of a project.

A deep engagement with patients may lead to better trust, understanding, and adoption of AI technologies in healthcare.^42^ Outreach can also help humans build trust in machines. This may enable better human-machine co-operation and adoption of AI in healthcare. Moreover, an effective way to build trust and understand a complex model (like an AI model) is to actively construct it.^31^^,^^32^

### Trust in AI and the role of PPI

There is a lot of discussion on how to build trust in AI models.^43^^,^^44^ Social and institutional factors are important in building trust.^15^

There is also a push toward trusted research environments that protect data and enable privacy-preserving analysis. Computational platforms like OpenSAFELY^45^ and federated analysis platforms like DataSHIELD^46^ can also enable computation while preserving privacy of individual-level patient data. Many projects have dedicated managers who are in charge of data governance.^47^ Communicating to patients that these roles exist and that there are people who look at data governance, ethics, and security may further bolster trust in the computational infrastructure.

Findability, accessibility, interoperability, and reusability (FAIR) principles are also important^48^ and should be explained to patients. Ethical and legal issues around the General Data Protection Regulation (GDPR), right to explanation,^49^ and duty of care can also be explained to patients in RAG meetings.

Patient involvement and public outreach are essential in facilitating ethical use of EHR data.^50^ Patients need to know the benefits of research using EHR data, which include answering questions about public health that would be unethical to pursue (for example, the effect of exposure to environmental toxins or inequalities in healthcare access).^50^

The public also lack understanding of how medical data can be used to improve healthcare.^51^ Educating patients and the general public about the benefits of research based on EHR data can help build trust.^50^

The general public does not fully trust clinical data sharing.^51^ However, attitudes become more positive once the benefits are explained to them.^51^

Adequately explaining AI research to patients and the public will help in getting a social license for research: research that is legitimate and compliant but does not have social license can be subject to constant challenge.^52^ This was manifested in the initiative to share general practitioner data in England (called care.data), which ultimately failed because of insufficient public outreach.^52^

As AI is incorporated in healthcare, involving patients and the public will help build confidence in these technologies.

Ethical considerations need to be embedded in the design and deployment of AI solutions.^53^^,^^54^ One of the ethical principles is multi-stakeholder collaboration.^54^ In the context of AI in healthcare, this means involving patients and carers in the design and deployment of AI tools.

AI practitioners in healthcare also need to think critically about the ethics of data, algorithms, and practices.^55^ The ethics of data focusses on data collection, re-identification risk, and privacy. The ethics of algorithms relates to the explainability of complex machine learning algorithms. Finally, the ethics of data science practice deals with unforeseen and undesired consequences of using machine learning algorithms.

Participatory design is an integral part of data science and data ethics.^56^ There is a need for researchers to think critically about the implications of their work and engage with the people who can be affected by their work.^56^ Engaging with patients and making them active co-producers of research will help data scientists continually reflect on the broader ethical and moral implications of their work. Patients should be made active participants in AI research on healthcare, as is being done in genomic research.^57^

Working closely with patients will help AI researchers reflect critically on the ethics of the algorithms they implement and remind them of their moral obligation to help patients. There is a need to critically reflect, at each step of the scientific process, on the ethical and moral implications of AI research in healthcare. It has been suggested that such continual reflection should become best practice in data science.^56^

As AI becomes more regulated, AI researchers should be incentivized to have an (at least informal) moral code of conduct and be cognizant of the impact of their work on patients. This can also be achieved by working in close partnership with patients, the ultimate beneficiaries of research. Guidelines for PPI by the National Institute for Health and Care Excellence (NICE) are a step in the right direction.^58^

AI researchers have a moral obligation to explain to patients how their data can help them. This has been called the duty of easy rescue.^50^ It has been argued that we have a moral and ethical responsibility to help other people, if this action causes little harm or discomfort to us.^59^

Participative design has also been used in other domains, like sustainability science, human-wildlife co-existence,^60^ and social infrastructure design.^61^ PPI has been used in co-designing complex mathematical models of infectious diseases.^62^ This can be used to build public confidence in modeling predictions and policies since research is co-produced with members of the public. Co-development is also used in transformative technologies, like gene drives for eradicating malaria, where the broader societal implications need to be debated and community support needs to be mobilized.^63^

Co-defining problems with stakeholders is important in other domains as well, like public and social sector organizations. Co-design has been used to reform the education sector, reduce social isolation in adults with cognitive disabilities, and restore children from foster care to birth families.^18^

Blindly promoting adoption of AI without consideration of the impact of these technologies can be detrimental. The European Commission has suggested co-design and public consultation as key components to build trust in future AI systems.^64^ Tighter regulations have been proposed in AI,^65^ as AI gets employed in human sentiment and emotion analysis,^66^ border control based on facial recognition,^67^ and recidivism prediction.^68^^,^^69^ Co-designing and actively involving the general public will help build trust in future AI applications.

Some principles for trustworthy AI have been proposed by the European Commission and are based on fundamental rights outlined in the European Union Charter of Fundamental Rights.^70^ They are (1) respect for human autonomy, (2) prevention of harm, (3) fairness, and (4) explainability. AI practitioners need to reflect on these principles and the broad implications of their work.

There is inadequate and imbalanced stakeholder representation in many of these discussions of AI.^70^ Citizens and representatives from civil society are absent in discussions of AI technologies in Europe.^70^

Many have suggested that AI poses systemic risks.^71^ One way to mitigate these risks in healthcare is to increase stakeholder (patient and carer) participation.

Reporting guidelines and checklists have been proposed for clinical trials that include AI.^72^ Such guidelines ensure reproducibility of AI methods applied to healthcare. We suggest that PPI and outreach should also be an integral component of checklists and guidelines for AI research in healthcare. A recent review has found that most studies do not involve co-design with patients, even though it is a critical component of digital health interventions.^73^

### Concluding remarks

In this work we have outlined case studies and a methodology of how modern data science can be applied to healthcare using a participatory design loop, where data scientists, clinicians, and patients work together. We have shared some best practices and tools that can be used for engaging with patients and explaining AI to them.

The strategy of co-designing AI algorithms with patients is a balanced approach. This can help set more realistic expectations for all stakeholders since conventional narratives of AI revolve either around dystopia or limitless optimism. We hope that AI research in healthcare can be adopted faster if humans slowly build up trust in machines, over repeated and carefully calibrated interactions.

The approach outlined here may have implications for mitigating risk and misinformation about AI in healthcare. Patients, data scientists, and healthcare workers can work together, thus benefiting patients.

## Ethics committee approval

The CPFT Research Database operates under UK NHS Research Ethics approvals (REC references 12/EE/0407, 17/EE/0442).
